# Randomized clinical trial of low dose suramin intravenous infusions for treatment of autism spectrum disorder

**DOI:** 10.1186/s12991-023-00477-8

**Published:** 2023-11-06

**Authors:** David Hough, Alice R. Mao, Michael Aman, Reymundo Lozano, Constance Smith-Hicks, Veronica Martinez-Cerdeno, Michael Derby, Zachary Rome, Niel Malan, Robert L. Findling

**Affiliations:** 1 PaxMedica, Inc, sponsor of the study, 303 S Broadway, Suite 125, Tarrytown, NY 10591 USA; 2 Psychiatry and Behavioral Sciences, Baylor College of Medicine and PaxMedica, Inc., Houston, Texas, USA; 3 O.S.U. Research Unit on Pediatric Psychopharmacology (RUPP), Research, Nisonger Center at The Ohio State University, Columbus, Ohio, USA; 4 Genetics and Genomics Sciences, Psychiatry and Pediatrics, Icahn School of Medicine, Mount Sinai, New York, NY USA; 5 Neurology, Kennedy Krieger Institute, Johns Hopkins Univ School of Medicine, Baltimore, MD, USA; 6 Pathology and Laboratory Medicine, Anatomy of Autism and FXS laboratory, Institute for Pediatric Regenerative Medicine, Shriners Hospital of Northern California, and UCD MIND Institute. Pathology Director of the FXS & FXTAS brain repository at UCD, University of California Davis School of Medicine, Davis, California, USA; 7 Tardimed Sciences LLC., PaxMedica, Inc., Tarrytown, New York, USA; 8 PaxMedica, Inc., Tarrytown, New York, USA; 9 Phoenix Pharma LTD, 2 Eastbourne Rd, Mt Croix, Port Elizabeth, South Africa; 10 Chair, Department of Psychiatry, Virginia Commonwealth University School of Medicine, Richmond, Virginia, USA; 11 Director of Fragile X Syndrome Clinical at Mount Sinai Hospital, New York City, New York, USA

**Keywords:** Autism spectrum disorder, Suramin, Purinergic receptor antagonist

## Abstract

**Background:**

There is a critical need for effective treatment of the core symptoms of autism spectrum disorder (ASD). The purinergic antagonist suramin may improve core symptoms through restoration of normal mitochondrial function and reduction of neuro-inflammation via its known antagonism of P2X and P2Y receptors. Nonclinical studies in fragile X knockout mice and the maternal immune activation model support these hypotheses.

**Methods:**

We conducted a 14 week, randomized, double-blind, placebo-controlled proof -of-concept study (N = 52) to test the efficacy and safety of suramin intravenous infusions in boys aged 4–15 years with moderate to severe ASD. The study had 3 treatment arms: 10 mg/kg suramin, 20 mg/kg suramin, and placebo given at baseline, week 4, and week 8. The Aberrant Behavior Checklist of Core Symptoms (ABC-Core) (subscales 2, 3, and 5) was the primary endpoint and the Clinical Global Impressions—Improvement (CGI-I) was a secondary endpoint.

**Results:**

Forty-four subjects completed the study. The 10 mg/kg suramin group showed a greater, but statistically non-significant, numeric improvement (− 12.5 ± 3.18 [mean ± SE]) vs. placebo (− 8.9 ± 2.86) in ABC-Core at Week 14. The 20 mg/kg suramin group did not show improvement over placebo. In exploratory analyses, the 10 mg/kg arm showed greater ABC Core differences from placebo in younger subjects and among those with less severe symptoms. In CGI-I, the 10 mg/kg arm showed a statistically significant improvement from baseline (2.8 ± 0.30 [mean ± SE]) compared to placebo (1.7 ± 0.27) (p = 0.016). The 20 mg/kg arm had a 2.0 ± 0.28 improvement in CGI-I, which was not statistically significant compared to placebo (p = 0.65).

**Conclusion:**

Suramin was generally safe and well tolerated over 14 weeks; most adverse events were mild to moderate in severity.

*Trial Registration* Registered with the South African Health Authority, registration number DOH-27–0419-6116. ClinicalTrials.Gov registration ID is NCT06058962, last update posted 2023–09-28.

## Background

Autism spectrum disorder (ASD) is a complex neurodevelopmental disorder with a constellation of symptoms that usually presents in the first few years of life [1]. The core symptoms of ASD include difficulty in social communication and interactions, restricted interests, repetitive behaviors, and diminished functioning in social settings, school, and other areas of life [2]. Recent data from the Centers for Disease Control Morbidity and Mortality Weekly Report indicate that ASD affects approximately 1 in 36 children [3]. ASD is commonly associated with many other symptoms including sleep disturbances, anxiety, depression, attention deficit symptoms, seizures, cognitive impairment, sensitivity to sensory inputs, gastrointestinal disturbances, and irritability. The core and associated symptoms have a significant impact on quality of life for individuals with ASD as well as their family members and caregivers [3]. FDA has approved two medications for the “treatment of irritability associated with autistic disorder” (Risperdal^®^ [risperidone] and Abilify^®^ [aripiprazole] USPI); however, the FDA has not approved any medicine for the treatment of core symptoms of the disorder. Current treatment focuses on behavioral therapy, educational interventions, and medicine to treat specific symptoms such as irritability, sleep disturbances, anxiety, or attention deficit symptoms [3]. There is a critical unmet need for effective treatment for the core symptoms of ASD.

The pathophysiology of ASD is not known. ASD is a heterogenous disorder that likely has many different etiologies. The most prevalent opinion is that it originates from a combination of genetic and environmental factors that adversely affect neurodevelopment and lead to a clinical presentation with a wide range of symptoms and severities [1, 4]. Recent studies have implicated mitochondrial dysfunction as a potential key neurobiological mechanism for the disorder [5–8]. Mitochondria play a critical role in cellular functioning including energy production, cellular metabolism, intracellular calcium signaling, generation of reactive oxygen species (ROS), apoptosis, and regulation of innate and adaptive immunity [6]. Mitochondria, which are essential in meeting the brain’s high energy demands, are involved in neurodevelopmental processes such as neural stem cell proliferation, cell differentiation, cell maturation, formation of dendritic processes, and synaptic plasticity [6].

One body of research has examined the role of inflammation in the development of ASD. A Maternal Immune Activation (MIA) mouse model of ASD generated by exposing female mice to a simulated viral infection by injection of double-stranded RNA poly (Inosine: Cytosine) during pregnancy determined that MIA dams produce offspring with symptoms that are similar to those of children with ASD. These symptoms include deficient social and communicative behaviors, as well as high levels of repetitive behaviors [9, 10]. Pardo and colleagues demonstrated neuroglia and innate immune system activation in brain tissue and cerebral spinal fluid (CSF) of individuals with ASD [11, 12]. Theoharides and colleagues proposed that activation of mast cells in the central nervous system (CNS) may lead to mitochondrial fission and translocation to the cell surface where they secrete ATP and DNA to the extracellular space [13]. ATP and DNA may be misconstrued by the body as “innate pathogens” leading to a strong autoimmune response and to neuro-inflammation [13].

Suramin is an anti-trypanosomal and anti-purinergic agent that was introduced in 1923 to treat T.b. *rhodesiense* Human African Trypanosomiasis, also known as East African Sleeping Sickness [14]. It is a polysulfonated naphthylurea compound that remains in the body for a prolonged period of time due to its stability, long half-life of 40–60 days, and 99.7% affinity for serum proteins [15–17]. Suramin acts as an antagonist at most purinergic receptors including P2Y and P2X receptors, which are widely distributed throughout the CNS. P2X and P2Y receptor antagonists may help reduce extracellular ATP and restore normal mitochondrial functioning [18]. Suramin is a potent anti-inflammatory agent, which may be related to its ability to block purine receptors [19, 20].

Animal models of ASD provide valuable nonclinical tools to investigate potential hypothesis-driven treatments for the disorder [21, 22]. Purine receptor antagonists produce symptomatic improvements in the core symptoms of autism in fragile X messenger ribonucleoprotein 1 (FMR1) knock-out mouse models [23]. PaxMedica has conducted a series of 5 nonclinical studies of suramin and other anti-purinergic compounds in FMR-1knockout mice. The results suggest that anti-purinergic receptor medications may restore normal short-term memory, social activity, and normal exploratory activity, which are typically absent in this FMR-1 transgenic mouse model (Company data on file). Naviaux and colleagues demonstrated that in the maternal immune activation mouse model of neurodevelopment, a single dose of suramin 20 mg/kg reversed disturbances in social behavior, novelty preference, and purine metabolism [10, 24]. Naviaux et al. tested suramin in a small pilot study (n = 10) in boys with ASD reporting safety and tolerability of a single 20 mg/kg dose and symptomatic improvement in language, social interaction, and decreased restricted or repetitive behaviors versus placebo [25]. Autism Diagnostic Observation Schedule (ADOS)‐2 comparison scores improved by − 1.6 ± 0.55 points (p = 0.0028) in the suramin group and did not change in the placebo group [25]. The current study builds upon this previous work supporting suramin as a potential treatment for core symptoms of ASD.

## Methods

### Study design

We conducted a proof-of concept, prospective, randomized, double-blind, placebo-controlled, multicenter, dose-ranging study of 2 doses of suramin (10 mg/kg and 20 mg/kg) versus placebo in 52 boys with ASD, ages 4–17 years. The primary objectives of the study were the safety, tolerability, and efficacy of suramin in children with autism. Investigators confirmed the diagnosis and that each subject had at least moderate ASD symptoms, based on the ADOS-2 comparison score.

The Aberrant Behavior Checklist (ABC) is an informant rating scale that is widely used in pharmacological research; it has well-established reliability, validity, and drug sensitivity [26–28]. Its five subscales are 1 (irritability, agitation, crying); 2 (lethargy/social withdrawal), 3 (stereotypic behavior), 4 (hyperactivity/noncompliance); and 5 (inappropriate speech). Prospectively, we designated improvements in the sum of ABC subscales 2, 3, and 5, the ABC-Core*,* as our primary endpoint. The ABC-Core has not previously been used as a singular outcome variable, although the three separate subscales have been used extensively in drug research. Subscales 1 and 4 were not included in ABC-Core as these subscales did not assess core ASD symptoms, but they were analyzed separately.

Secondary endpoints included ABC-Total Score (including all 5 subscales), Clinical Global Impression of Improvement (CGI-I) adapted for autism, Autism Treatment Evaluation Checklist (ATEC), and Expressive One Word Picture Vocabulary Test (EOWPVT). All primary and secondary endpoints were prespecified in the statistical analysis plan. A post hoc analysis was conducted with the ABC-Core evaluating the impact of age and severity of illness at baseline on efficacy outcomes.

**Fig. 1:**
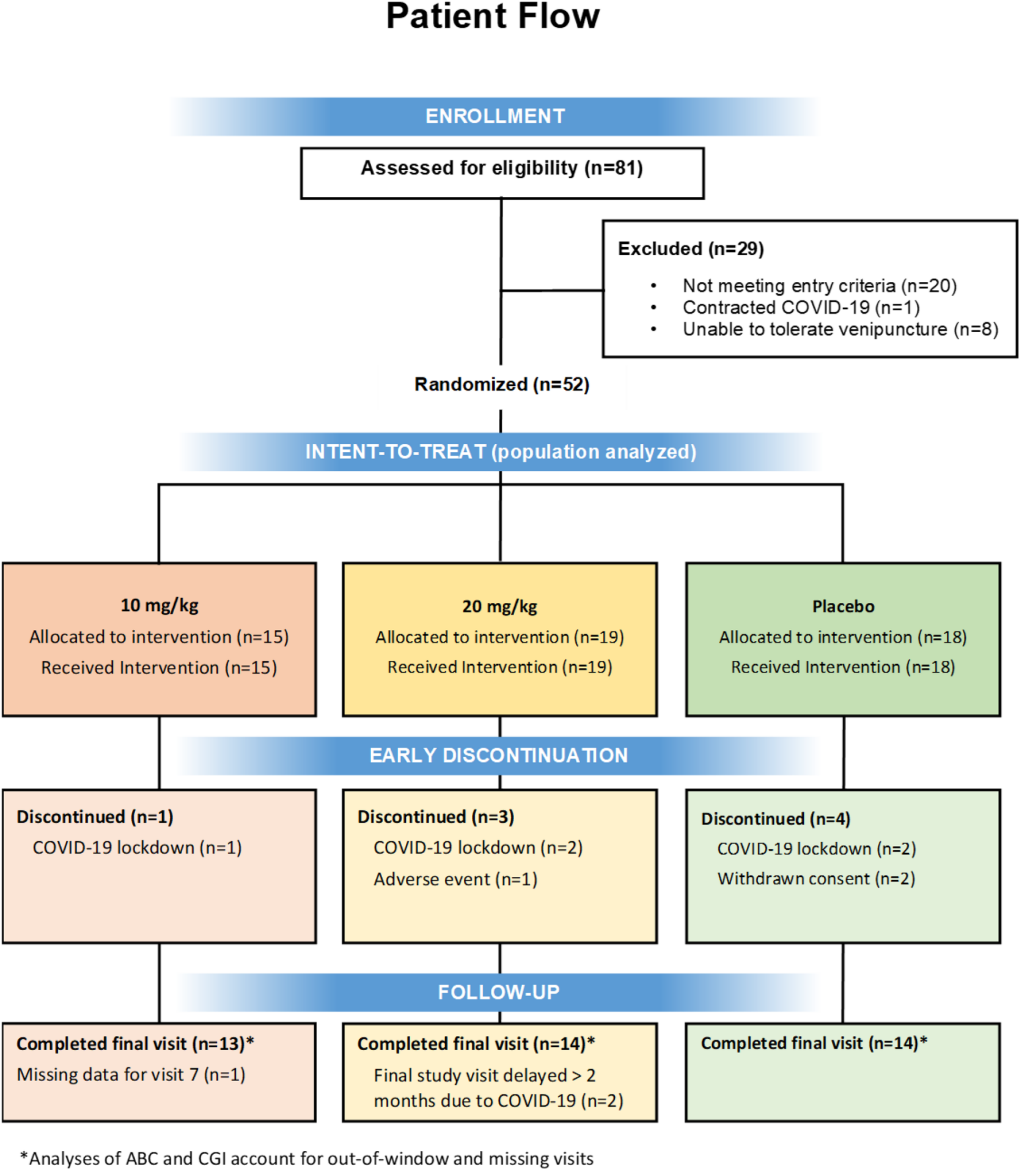
Patient flow

**Fig. 2:**
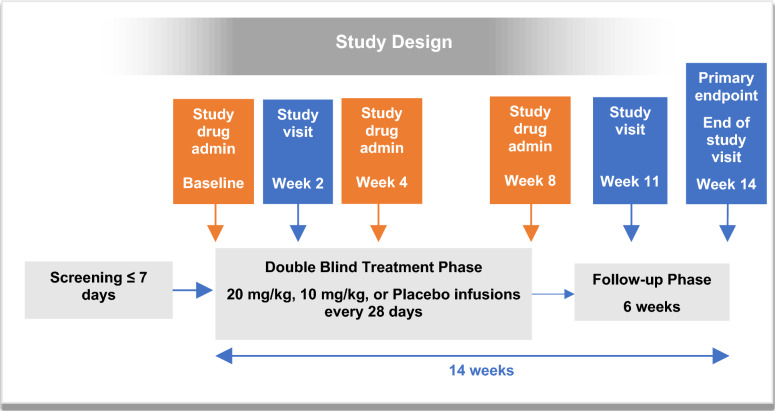
Study design

There were 3 intravenous infusion treatment groups: suramin 20 mg/kg, suramin 10 mg/kg, and placebo. Treatment was administered at baseline, week 4, and week 8. The higher dose was chosen based on the previous Naviaux et al., 2017 study. This study used a single dose of 20 mg/kg, which was well tolerated, and some efficacy benefits were observed in 5 participants with ASD. A lower dose of 10 mg/kg was also chosen to determine if a lower dose would show similar efficacy and potentially better safety and tolerability. Total duration of the study was 14 weeks. The details of the patient flow and study design are shown in Figs. 1 and  2, respectively. The study was conducted at 6 sites in South Africa, where suramin is a registered medicine and was approved by the South African Health Products Regulatory Authority and the National Health Research Ethics Council on February 19, 2019 (Application 3DOH-27–0419-6116). The ClinicalTrials.Gov ID is NCT06058962. Each of these sites were outpatient treatment centers and subjects were recruited through local advertising. Each family member or caregiver was given a small stipend to cover out of pocket expenses (e.g., transportation, meals) for each study visit. The amount of these stipends was reviewed and approved by local ethics committees. The study was conducted according to the ethical principles of the Declaration of Helsinki, International Conference on Harmonization guidelines for Good Clinical Practices (GCP).

Inclusion criteria included males aged 4 to 17 years with a diagnosis of ASD by Diagnostic and Statistical Manual of Mental Disorders, 5th edition (DSM-V), ADOS-2 comparison scores in the moderate and high level as evaluated on the ADOS-2, and stable treatment intervention for ≥ 2 months. Participants agreed to remain on a stable treatment intervention throughout the study and participants on methylphenidate and risperidone or similar medication agreed to maintain a stable dose during the study.

Exclusion criteria included psychiatric hospitalization within the previous 2 months, an acute medical problem, Rett syndrome, microcephaly, tuberous sclerosis, neurofibromatosis, epilepsy or children with known syndromic forms of ASD caused by DNA mutation or chromosomal copy number variation. Other exclusion criteria included any clinically significant liver, kidney, or adrenal disease, serious acute condition, plans to start a new drug, diet, or behavioral intervention during the study, weight under the 5th percentile for age, plasma creatinine above normal for age and weight according to the laboratory reference ranges, liver enzyme alanine aminotransferase (ALT) or aspartate aminotransferase (AST) ≥ 1.5-fold above the upper limit of normal, and known intolerance to suramin or other antipurinergic drugs.

The study was conducted between May 2019 and December 2020. There was a pause of approximately 6 months during the COVID-19 pandemic as clinics closed and families were unwilling to come in for clinical visits. This resulted in five participants dropping out of the study (Fig. 1). The sample size was increased to 52 (48 originally planned) to replace these early withdrawal participants.

### Statistical analysis

Statistical analysis was governed by a Statistical Analysis Plan (SAP), which was amended on 26 January 2020 to accommodate delays in study visits due to COVID-19. The Intention-to-Treat (ITT) population, which was the primary efficacy analysis population, consisted of all randomized participants. The sample size of 52 randomized 1:1:1 was chosen to yield 80% power to detect a difference of 2 units between treatment arms with a significance level of 0.05. This calculation was based on a between-participant standard deviation based on the ABC – Total Score of about 2.3 in suramin and 4.3 in placebo as reported by Naviaux et al., 2017 [25].

After signing the informed consent and assent forms, the participants were allocated a 3-digit participant number that was used to identify them throughout the study. In each center, participant numbers were assigned in sequential order. The site requested the central randomizer to randomize the participant. Each participant was assigned to one of the three double-blind treatment groups for the duration of the study. The randomization was also stratified according to Age, ADOS-2 and Non-verbal Intelligence Quotient (NVIQ) as assessed by the Leiter International Performance Scale, 3rd Edition (Leiter-3). Participants were randomized to one of three double-blind treatment groups, i.e., Arm A (10 mg/kg suramin) or Arm B (20 mg/kg suramin) or Arm C (placebo) in a targeted 1:1:1 ratio, as per the randomization schedule and stratification plan. The stratification plan was to match patients by age (< 7 vs ≥ 7), ADOS-2 comparison scores (≤ 8.5 vs > 8.5) and NVIQ (≤ 80 vs > 80).

For efficacy modeling, the SAP approach for missing data would focus on the ABC-Core and CGI outcomes for participants missing Week 14 data resulting from withdrawal, drop-out, loss-to-follow-up, or missed visits. Because of COVID quarantine, the planned approach for the ABC-Core was to apply a wide window to the last two visits, and to use a single imputation. The CGI-I was recorded at each timepoint relative to the previous one; the sum of all timepoints represents the week 7 change from baseline. The approach for CGI-I missing data was to use all available timepoint data, which is akin to a last-observation-carried-forward (LOCF) approach and assumes no additional changes after the last timepoint for non-completers. An analysis of variance (ANOVA) used changes at Week 14 compared to baseline for ABC-Core and Week 14 scaled scores (as described above) for CGI-I as responses, with categorical treatment group and baseline age, ADOS-2, and non-verbal IQ (NVIQ) (all continuous) as covariates. P-values for ABC-Core and CGI analyses used Dunnett’s method for multiple comparisons. As this study was aimed at selecting viable outcomes for future studies, there were no other adjustments for multiple outcomes.

### Pharmacokinetic analysis

Pharmacokinetic samples were obtained at seven time points: Baseline (before and 1 h after infusion), day 28 (before and 1 h after infusion), day 56 (before and 1 h after infusion), and at the end of the study (day 98). PK plasma samples with lithium heparinate as anticoagulant were collected and stored at ~ − 20 °C and analyzed after the study by Farmovs Integrated Research Solutions in Bloemfontein, South Africa. Extraction from the biological matrix was performed with a protein precipitation technique, liquid chromatography with tandem mass spectrometry detection (LC–MS/MS) Sciex API4000. The software used Watson LIMS^™^ software version 7.4.2 and Analyst^®^ software version 1.6.2.

## Results

A diverse sample of 52 boys between 4 and 15 years (mean [SD] 7.9 [3.2] years) was randomized. Baseline demographics and assessment scores for ADOS-2 and Leiter-3 are shown in Table 1. The average age was 6.9 years in the 10 mg/kg group, 8.9 years in the 20 mg/kg group, and 7.8 years in the placebo group. Racial composition was Black (n = 22), White (n = 20), Mixed Race (n = 8), and Asian (n = 2). The mean ADOS-2 scores at baseline were 8.1 for the10 mg/kg group, 8.3 for the 20 mg/kg group, and 8.1 for the placebo group. The mean Leiter-3 scores at baseline were 68.3 for the 10 mg/kg group, 67.6 for the 20 mg/kg group, and 67.4 for the placebo group. The most frequent concomitant medications at baseline were risperidone (n = 5) for irritability associated with ASD and methylphenidate (n = 5) for ADHD.

**Table 1:** Demographics at baseline

	*Placebo* *N* = *18*	*10 mg* *N* = *15*	*20 mg* *N* = *19*	*Total* *N* = *52*
Age (years)				
N	18	15	19	52
Mean (SD)	7.8 (3.1)	6.9 (2.2)	8.9 (3.7)	7.9 (3.2)
Range (min, max)	4, 13	4, 11	4, 15	4, 15
Quartiles (25th, median, 75th)	5, 8, 10	5, 7, 8	6, 8, 11	5, 8, 10
Race, n (%)				
Black	7 (39)	8 (53)	7 (37)	22(42)
White	7 (39)	5 (33)	8 (42)	20(38)
Mixed race	3 (17)	2 (13)	3 (16)	8(15)
Asian	1 (6)	0	0	1 (2)
Indian	0	0	1 (5)	1 (2)
Weight (kg)				
N	18	15	19	52
Mean (SD)	30.55 (10.15)	27.57 (7.32)	38.23 (16.97)	32.50 (13.09)
Range (min, max)	15.1, 49.2	18.6, 46.3	18.5, 81.4	15.1, 81.4
Quartiles (25th, median, 75th)	23.1, 29.2, 40.9	22.7, 26.1, 28.1	25.0, 32.2, 46.9	23.6, 29.0, 38.2
ADOS-2				
N	18	15	19	52
Mean (SD)	8.1 (1.5)	8.1 (1.3)	8.3 (1.4)	8.2 (1.4)
Range (min, max)	6, 10	6, 10	6, 10	6, 10
Quartiles (25th, median, 75th)	7, 8, 9	7, 8, 9	7, 8, 10	7, 8, 9
**NVIQ**				
N	18	15	19	52
Mean (SD)	67.4 (26.2)	68.3 (29.6)	67.6 (29.0)	67.7 (27.6)
Range (min, max)	30, 124	30, 119	30, 112	30, 124
Quartiles (25th, median, 75th)	39, 71, 85	39, 65, 100	39, 75, 93	39, 72, 88

Column header counts and denominators are the number of subjects in the ITT population

**Fig. 3:**
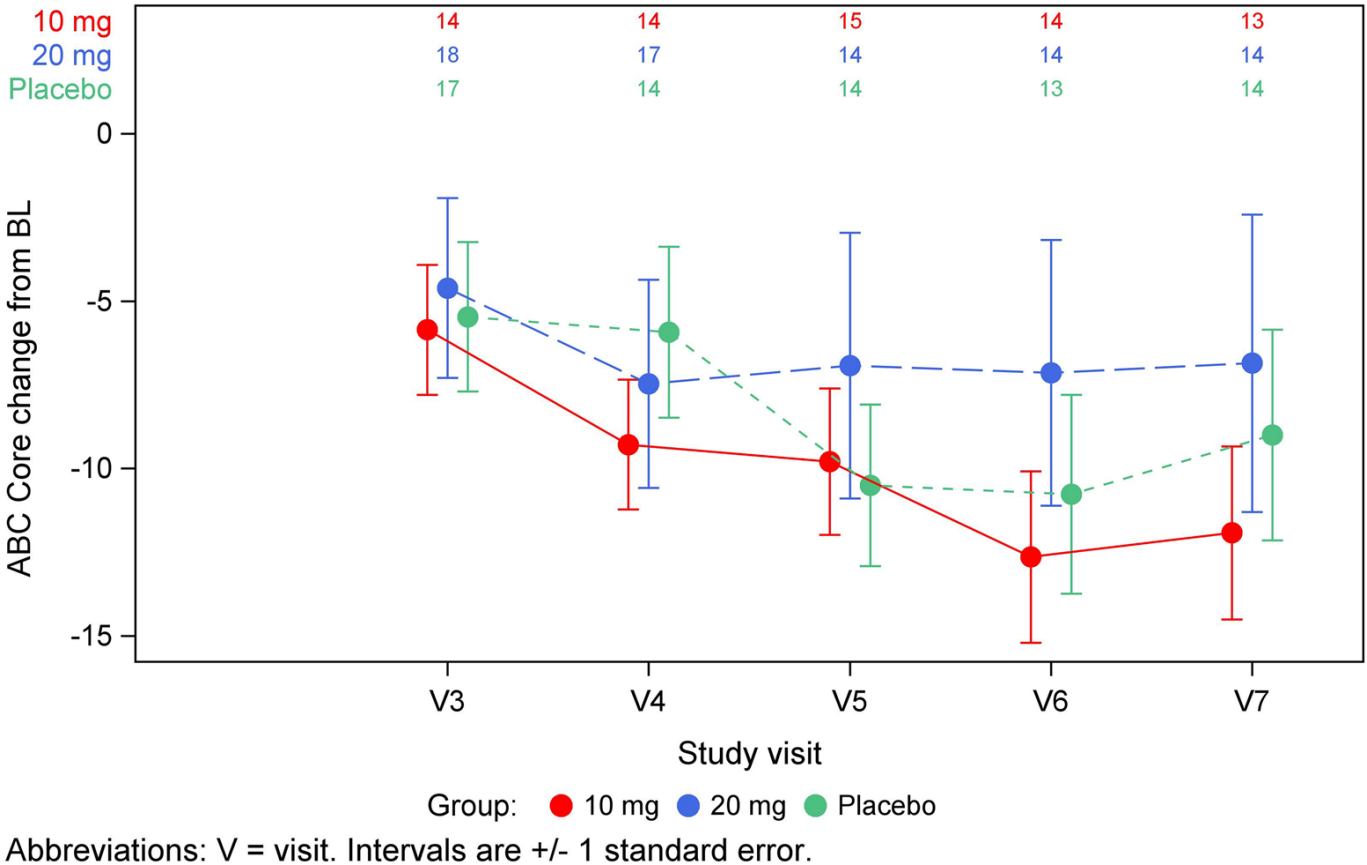
ABC-Core change from baseline by dose group over time

Forty-four of the 52 subjects completed the trial. The most common cause for early discontinuation was COVID-19 quarantine and associated site closure (n = 5). One subject was discontinued because of an adverse event and two for withdrawal of consent. The results of the primary endpoint, improvement in ABC-Core, are shown in Fig. 3 and the results of all primary and secondary efficacy assessments are shown in Table 2. For the primary endpoint of ABC-Core, the 10 mg/kg dose group had a 12.3-point decrease from baseline vs. an 8.4 point decrease for placebo, but the difference was non-significant, p = 0.37 (unadjusted) and p = 0.58 (adjusted). The 20 mg/kg dose group did not show any improvement after week 4 and was not significantly different than placebo.

**Table 2:** Modeled efficacy results comparing active treatments with placebo

Dose Group (n)	ABC-Core^a^	ABC-Total^a^	CGI-I Question 1^bc^	CGI-I 24 item^bc^	ATEC^ac^
10 mg/kg (13)					
Mean change from BL ± SE	− 12.3 ± 3.25	− 32.0 ± 6.88	2.8 ± 0.30	73.4 ± 5.57	− 20.9 ± 4.09
Difference from placebo:					
Mean change from BL (95% CI)	− 3.9 (− 13.9, 6.1)	− 14.7 (− 35.8, 6.3)	1.1 (0.2, 2.0)	21.2 (4.2, 38.3)	− 4.3 (− 17.5, 9.0)
P value, unadjusted	0.37	0.12	0.008	0.007	0.46
P-value, adjusted^d^	0.58	0.20	0.016	0.013	0.68
20 mg/kg (14)					
Mean change from BL ± SE	− 6.5 ± 2.89	− 16.2 ± 6.13	2.0 ± 0.28	59.5 ± 5.11	− 15.2 ± 4.04
Difference from placebo:					
Mean change from BL (95% CI)	2.0 (− 7.5, 11.4)	1.1 (− 18.9, 21.0)	0.3 (− 0.6, 1.2)	7.3 (− 9.1, 23.7)	1.5 (− 12.0, 14.9)
P value, unadjusted	0.64	0.90	0.43	0.31	0.80
P-value, adjusted^d^	0.85	0.99	0.65	0.50	0.96
Placebo (14)					
Mean change from BL ± SE	− 8.4 ± 2.91	− 17.3 ± 6.17	1.7 ± 0.27	52.2 ± 4.99	− 16.6 ± 4.07

*ABC-Core* aberrant behavior checklist of core symptoms, *ABC-Total*, aberrant behavior checklist—total score, *ATEC*  autism treatment evaluation checklist, *BL* baseline, *CGI-I* question 1, clinical global impression of improvement question 1 overall severity of symptoms, *CGI-I* 24 item, summary score of clinical global impression of improvement for all 24 items; *CI* Confidence Interval, *SE* standard error

^a^Negative score indicates improvement

^b^Positive score indicates improvement

^c^Nominal p-values

^d^Via Dunnett’s method

For ABC-Total Score, the 10 mg/kg dose showed a consistent improvement throughout the 14 week study and a nonsignificant separation of 14.7 points from placebo (p = 0.12, unadjusted and p = 0.20, adjusted). The 20 mg dose did not show improvement after week 4 and was not significantly different than placebo.

The CGI-I was a secondary endpoint. The statistical analysis plan focused on the overall severity of symptoms, which showed a mean improvement from baseline of 2.8 points for 10 mg/kg, 2.0 points for 20 mg/kg, and 1.7 points for placebo. The improvement in CGI-I for the 10 mg/kg dose compared to placebo was statistically significant (p = 0.008, unadjusted and p = 0.016, adjusted).

The Autism Treatment Evaluation Checklist (ATEC) was also a secondary endpoint. In the ITT Population, the ATEC-Total outcome score means (SD) change from baseline to Visit 7 (Week 14) was -20.9 (4.09) for the 10 mg/kg suramin group, − 15.2 (4.04) for the 20 mg/kg suramin group, and -16.6 (4.07) for the placebo group (see Table 2). The 10 mg/kg showed slightly greater numeric improvement compared with both placebo and suramin 20 mg/kg; however, the results were not statistically significant.

**Fig. 4:**
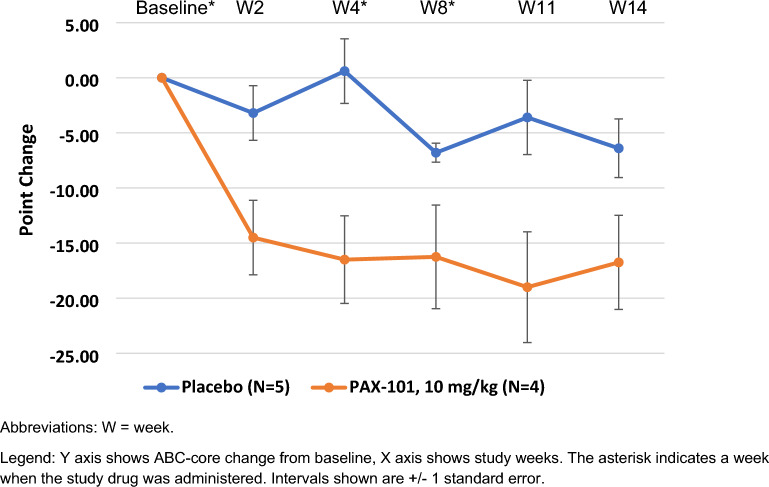
Exploratory analysis of ABC-Core in participants with less severe symptoms at baseline (ADOS 6–7) treated with 10 mg/kg or placebo

**Fig. 5:**
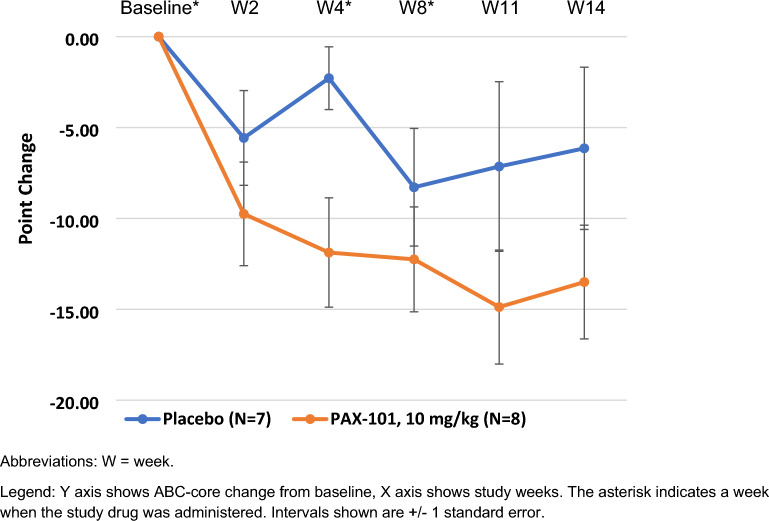
Exploratory analysis of ABC-Core in younger participants (age < 8 years) treated with 10 mg/kg or placebo

We conducted several exploratory analyses of the primary endpoint, ABC Core, in patients treated with 10 mg/kg to identify subpopulations that experienced a greater treatment effect. We noted that subjects with less severe symptoms, with ADOS comparison scores at baseline of 6 or 7, Fig. 4, and subjects who were younger than 8 years of age, Fig. 5, showed a greater improvement than the overall group, Fig. 3 and Table 2.

Assessment of safety and tolerability of suramin over 14 weeks of treatment were important objectives for this study. The adverse events occurring in 3 or more subjects are shown in Table 3. The most common TEAE was rash, upper respiratory tract infection, decreased white blood cell count, vomiting, aggression, pyrexia, constipation, and decreased appetite. One serious adverse event occurred in the study. The subject was a 4 year-old boy with cerebral palsy, hydrocephalus, and a ventriculoperitoneal shunt. A reported event of status epilepticus occurred 26 days after his second infusion of 20 mg/kg. He recovered without sequelae, was discharged from the hospital on oral valproic acid, and discontinued from the study. The investigator classified the event as severe and possibly related to study medication. One other subject was discontinued from further treatment due to a general body rash, which the investigator assessed as possibly related to the study medication. No clinically significant abnormalities were observed in lab, vital signs, or physical examinations.

**Table 3:** Adverse events

Preferred Term	Placebo N = 18	10 mg/kg N = 15	20 mg/kg N = 19	Total N = 52
Any AE n (%)	11 (61)	9 (60)	16 (84)	36 (69)
Rash^a^	5	1	8	14
Upper respiratory tract infection	4	3	3	10
Decreased white blood cell count^b^	4	1	3	8
Vomiting	1	0	6	7
Aggression	0	3	2	5
Pyrexia	2	1	3	6
Constipation	1	1	1	3
Decreased appetite	1	1	1	3

^a^Including several similar terms such as rash, macular rash, and maculopapular rash

^b^including several similar terms such as leukopenia, lymphopenia, and neutropenia

The plasma concentrations 1 h after the end of infusion, trough concentration, and other pharmacokinetic parameters are shown in Table 4. These results should be interpreted with caution as they were sparse samples and did not capture the full elimination curve of suramin between the day of dosing and trough concentrations one month later.

**Table 4:** Observed plasma concentrations

Dose Group (mg/kg)		Parameter									
		Concentration 1 h after end of infusion						C_trough_			
		Day						Day			
		1		28		56		28		56	
		ug/mL	(ug/mL)/mg	ug/mL	(ug/mL)/mg	ug/mL	(ug/mL)/mg	ug/mL	(ug/mL)/mg	ug/mL	(ug/mL)/mg
**10**	N	15	15	15	15	15	15	15	15	15	15
	Mean	229	0.838	157	0.605	164	0.640	4.31	0.017	7.96	0.030
	Min	120	0.40	111	0.41	133	0.36	3.12	0.01	5.33	0.02
	Median	168	0.61	160	0.58	167	0.61	4.16	0.02	7.28	0.03
	Max	1150	3.52	189	0.84	200	0.90	7.07	0.02	11.4	0.04
	CV%	112	92.1	15.3	22.2	11.3	23.5	23.8	26.4	22.9	22.7
	Geometric mean	182	0.692	155	0.591	163	0.622	4.21	0.016	7.78	0.030
	CV% geometric mean	59.7	58.6	16.1	22.6	11.3	25.4	22.0	29.0	22.5	25.8
**20**	N	18	18	16	16	15	15	16	16	15	15
	Mean	328	0.51	327	0.53	332	0.52	11.0	0.018	21.1	0.033
	Min	214	0.15	193	0.12	172	0.13	6.16	0.00	7.60	0.01
	Median	344	0.52	330	0.54	353	0.58	11.3	0.02	24.40	0.04
	Max	439	0.92	470	0.97	459	0.77	14.3	0.03	30.20	0.05
	CV%	20.5	43.4	28.0	44.4	25.8	36.4	18.8	34.0	35.9	45.0
	Geometric mean	321	0.456	314	0.47	320	0.47	10.8	0.016	19.4	0.029
	CV% geometric mean	22.3	52.8	29.9	59.8	30.0	55.6	21.2	51.7	48.0	71.4

## Discussion

The design and purpose of this dose ranging, proof-of-concept study was to determine if multiple doses of suramin treatment over 14 weeks are safe and tolerable, and to determine possible efficacy for core symptoms of the disorder, as measured by ABC-Core. One of the two doses, 10 mg/kg, showed a greater, but statistically nonsignificant, improvement compared with placebo. The original power calculation was based on the results from a small number of subjects (n = 5 suramin and n = 5 placebo) on the ABC-Total score from the Naviaux 2017 study [25]. A larger sample size will be required to have sufficient power to detect a statistically significant difference on ABC-Core.

Secondary efficacy endpoints, such as ABC-Total score, CGI-I, and ATEC consistently showed that the 10 mg/kg dose had a greater numeric change from baseline than placebo (Table 2). The difference between the 10 mg/kg dose group and placebo was greater for the improvement in ABC-Total score than for the improvement in ABC-Core. This was due to the greater decrease in subscales 1 (irritability) and 4 (hyperactivity) than in the other subscales. The treatment effect for the 10 mg/kg dose compared to placebo for CGI-I was nominally statistically significant and the magnitude of the improvement (a 2.8-point increase from baseline) was clinically meaningful. Our post hoc analysis showed a greater improvement in ABC-Core in younger individuals and in individuals with less severe symptoms (Figs. 3, 4) than in the overall population (Fig. 3). We hypothesize that older subjects with more severe symptoms, mainly nonverbal, may have been more treatment resistant.

The Naviaux study included 10 boys, 5 were treated with a single dose of 20 mg/kg suramin and 5 with placebo. The ADOS-2 comparison scores and the Expressive One-Word Picture Vocabulary Test (EOWPVT) as the primary outcome assessments for efficacy. The ADOS-2 improved by -1.6 ± 0.55 points (n = 5; 95% CI − 2.3 to − 0.9; Cohen’s d = 2.9; P = 0.0028) in the suramin group and did not change in the placebo group. The EOWPVT did not change. We included the ADOS-2 in our eligibility criteria and stratification plan but chose to measure efficacy outcome based on the ABC core and total scores. We observed non-statistically significant efficacy improvements in the 10 mg/kg dose groups for the primary outcome assessment, ABC-Core as well as for the ABC Total Score and ATEC. We observed a statistically significant improvement in the secondary outcome measure, CGI-I.

Suramin infusion did not demonstrate a monotonic dose response for efficacy; compared with the 20 mg/kg dose group, the 10 mg/kg dose showed a greater change from baseline across multiple efficacy assessments. A nonlinear or inverted “U” dose response curve has been reported with several CNS medications and treatments such as tricyclic antidepressants, psychedelics, opioids, cannabinoids, and nicotine [29–32]. There are several potential hypotheses that might explain a non-monotonic dose response. Continuous receptor stimulation may result in downregulation or desensitization and lead to a diminished response. Given the long half-life of suramin (40–60 days) and the 4 week dosing interval, drug accumulation in the 20 mg/kg dose group may have contributed to this effect. Doses higher than necessary for an optimal clinical effect may lead to functional changes that interfere with the clinical improvements observed at lower doses. This phenomenon has been observed with dosing of D2 antagonists and neuroplasticity [31]. Off-target interactions at the receptor level may also contribute to a non-monotonic dose response. An example of this phenomenon is the anxiolytic effects of cannabinoids that have an inverted U-shaped dose–response curve in humans, which may involve off-target interactions with other CNS receptors [30].

TEAE data showed that most events were mild to moderate in severity, self-limited, and resolved spontaneously or with over-the-counter medications. All but one event occurred on the day of dosing.

The pharmacokinetic data in this study were limited due to sparse sampling and were not suitable for noncompartmental pharmacokinetic analysis. Cooper and colleagues described the concentration versus time profile for suramin in plasma after intravenous infusion using a 3-compartment model with rapid and slow disposition phases (half-lives 2.2 and 34.7 h, respectively) and elimination half-life of 1205 h (50.2 days) [33]. In our pediatric study, no samples were collected between the 4 week infusion intervals; therefore, the distribution phase and elimination half-life could not be estimated. Future studies with rich sampling will be required to determine the suramin half-life and total exposure in pediatric patients.

The study has several limitations. The sample size may have been too small for adequate statistical power to detect small, but important, differences between suramin and placebo. To assess efficacy, a study with a larger sample size is required. Because the study was 14 weeks in duration, we were not able to assess long-term safety or efficacy of suramin in this population. We have limited safety data for suramin in pregnancy and elected to limit the sample to boys as we planned to study pediatric subjects in the age range of 4 to 17 years. Older girls would be in an age range where pregnancy is a possibility. This prevents the generalizability of the results to girls with ASD.

## Conclusion

Monthly suramin intravenous infusions may be a safe and potentially efficacious treatment for the core symptoms of ASD.

## Data Availability

The source data for the study is maintained by the company and may be made public once the manuscript is published.

## Abbreviations

ABC: Aberrant behavior checklist
ADOS: Autism diagnostic observation scale
ANOVA: Analysis of variance
ASD: Autism spectrum disorder
ATEC: Autism treatment evaluation checklist
ATP: Adenosine triphosphate
CGI: Clinician global impression—improvement
CNS: Central nervous system
FDA: Food and Drug Administration
GCP: Good clinical practices
LC–MS/MS: Liquid chromatography with tandem mass spectrometry
MIA: Maternal immune activation
PK: Pharmacokinetic
ROS: Reactive oxygen species
TEAE: Treatment emergent adverse events
